# Evaluating the performance of a rapid antigen test for the detection of influenza virus in clinical specimens from children in Cameroon

**DOI:** 10.1111/irv.12210

**Published:** 2013-11-24

**Authors:** Sebastien Kenmoe, Patrice Tchendjou, Suzie Moyo Tetang, Tatiana Mossus, Mohammed Njankouo Ripa, Marlène Guillet, Anfumbom Kfutwah, Richard Njouom

**Affiliations:** a National Influenza Centre, Centre Pasteur of CameroonYaounde, Cameroon; b Université de Yaoundé IYaounde, Cameroon; c Centre Hospitalier d'EssosYaounde, Cameroon

**Keywords:** Cameroon, influenza, SD Bioline, sensitivity, specificity

## Abstract

The performance of SD Bioline rapid antigen test (RAT) was evaluated using real-time reverse transcription polymerase chain reaction (rRT-PCR) as gold standard. A total of 718 nasal swabs, including 102 rRT-PCR positive and 616 rRT-PCR negative swabs, were tested. RAT demonstrates a sensitivity of 29·4% with a specificity of 100%. The positivity rate of RAT was highly associated with lower cycle threshold (Ct) values (*P* < 0·0001). The excellent specificity of the RAT allowed for the rapid identification of influenza cases. However, negative results should be verified by rRT-PCR test because of limitations observed in sensitivity.

## Introduction

Rapid antigen tests (RATs) have become more frequently used as a tool for the surveillance of influenza activity.^1^,^2^ They are simple, can be performed out of the laboratory and provide rapid results within 30 minutes.^3^,^4^ One of the most sensitive methods for the identification of influenza is the real-time reverse transcription polymerase chain reaction (rRT-PCR)^5^,^6^; however, it requires specialized equipment and expertise which are unfortunately not available in primary healthcare settings in resource-limited countries. It is therefore more practical to use RAT to screen for influenza-like illnesses in clinics in such settings.

Reports from previous studies attest that these RATs have good specificity and moderate clinical sensitivity for use in the detection of seasonal influenza infections.^7^,^8^ However, the performance and utility of RAT under field conditions in resource-limited countries are not well described. We evaluated the sensitivity and specificity of the SD Bioline RAT by using the rRT-PCR as the standard reference.

From 23 September 2011 to 27 November 2012, a total of 718 children aged 0–15 years were prospectively enrolled in this study at the paediatric service of the National Social Insurance hospital, located at Essos in Yaounde, Cameroon. Clinical and demographic data were collected using case report forms. Samples were classified according to the symptoms presented, as influenza-like illness (ILI) or as severe acute respiratory infection (SARI). Age groups were stratified as follows: 0–1 years (G1), 1–5 years (G2) and 5–15 years (G3). Informed consent to participate in the study was obtained from the parents or legal guardians of the children. This study is part of a global study aimed at assessing risk factors associated with severe influenza. The study was approved by the National Research Ethics Committee and the Ministry of Health of Cameroon.

Nasal swabs were collected from patients admitted or consulting at the paediatric service of the National Social Insurance hospital for SARI or ILI. Samples collected at the patients' bedside or in the consultation room were split into two aliquots, and one was immediately tested by one of three practitioners with RAT (SD Bioline) in accordance with the manufacturer's instruction. The other aliquot was kept in a viral transport medium refrigerated at +4°C and later on during the day transported to Centre Pasteur du Cameroun (CPC) for rRT-PCR analysis. At the CPC, total RNA was extracted from 140 μl of each nasal swab sample using the QIAamp viral RNA minikit (QIAgen, Courtaboeuf, France) in accordance with the manufacturer's protocol. The samples were tested using a one-step rRT-PCR for the detection of influenza viruses A and B according to the US Centers for Disease Control and Prevention (CDC) protocol. Each positive rRT-PCR clinical specimen was characterized by their Ct value, with lower values indicating higher viral titres.

Sensitivity, specificity, positive predictive values (PPV) and negative predictive values (NPV) were calculated using standard formulas. The Fisher's exact test and the Student's test were used to compare the results in this study. Statistical analyses were performed using the R program version 2.15.1. A *P* value of 0·05 or less was considered to be statistically significant.

A total of 718 nasal swabs were collected and screened using rRT-PCR and the SD Bioline RAT. The median age of the participants was 1.5 years (IQR: 0·7–4), and 50% (359/718) of the samples were collected from male children. Among the 718 samples tested, 14·2% (102/718) were positive for influenza by rRT-PCR (Table 1). Of these 102 positive samples, 67·64% (69/102) were positive for H3N2, 24·50% (25/102) for type B and 7·84% (8/102) for 2009 H1N1 pandemic strain.

**Table 1:** Performance of the rapid antigen test SD Bioline compared with real-time reverse transcription polymerase chain reaction for the identification of influenza viruses

rRT-PCR for influenza	Rapid antigen test		Total
	Positive	Negative	
Positive	30	72	102
Negative	0	616	616
Total	30	688	718

rRT-PCR, real-time reverse transcription polymerase chain reaction.

**Figure 1:**
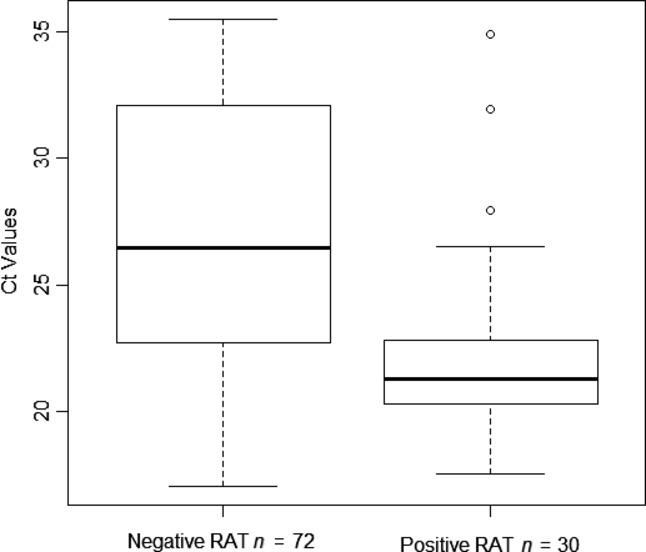
Comparison of cycle threshold (Ct) values for influenza real-time reverse transcription polymerase chain reaction positive specimens (*n* = 102) with negative and positive rapid antigen test (RAT) result. Within each box plot, the centre line represents the median, while the top and bottom borders mark the 75th and 25th percentiles, respectively. Whiskers represent the minimum and maximum values, except for an extreme outlier, which is indicated by an open circle. Boxplots were produced using the R program version 2.15.1.

The overall sensitivity of RAT was 29·4% (30/102). The sensitivity by age group was 45·7% (16/35) for G1, 27·9% (12/43) for G2 and 8·3% (2/24) for G3. The sensitivity by clinical presentation was 33·3% (23/69) for ILI patients and 21·2% (7/33) for SARI patients. The overall NPV of the RAT was 89·5%. It was 92·9, 89·0 and 84·1% for the G1, G2 and G3, respectively, and 89·0 and 90·4% for the ILI and SARI patients, respectively. There was a significant association between low Ct values by rRT-PCR (corresponding to higher concentrations of viral RNA) and sensitivity of RAT. Among positive samples in rRT-PCR, The mean Ct values of RAT negative specimens were significantly higher than those of RAT positive specimens (27·0 with 95% CI: 25·8, 28·2 versus 22·4 with 95% CI 21·0, 23·7, *P* < 0·0001) (Figure 1). There was a significant implication in the sensitivities of the RAT between the age groups, 45·7, 27·9, and 8·3% for G1, G2 and G3, respectively (*P* = 0·05). With regard to the clinical presentation, there was no implication in sensitivity between ILI cases and SARI cases (33·3 versus 21·2%, *P* = 0·37). The specificity and PPV were 100% for all groups.

**Figure 2:**
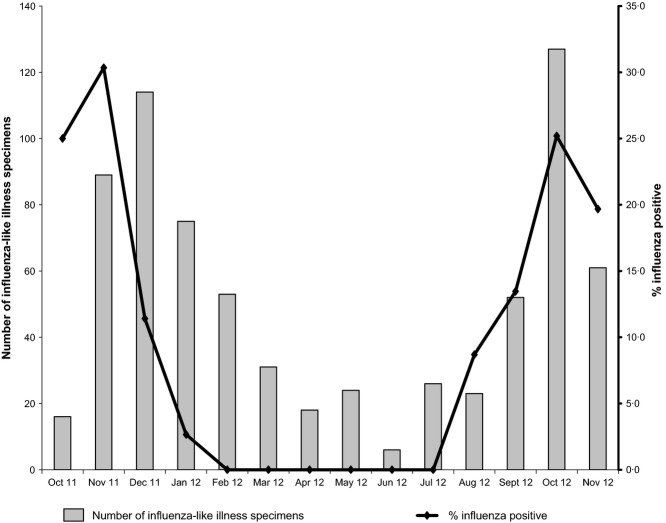
Number of influenza-like illness specimens (bars) and the rate of influenza positive specimens (line) from October 2011 to November 2012 in Yaounde, Cameroon.

Figure 2 presents the number of influenza-like illness specimens and the rate of influenza positive specimens from October 2011 to November 2012 in Yaounde, Cameroon. As reported in two previous studies in Cameroon,^9^,^10^ our study shows a clear seasonal trend of the circulation of influenza viruses in Cameroon with peaks occurring from October to November. There was a low rate of influenza circulation from January to July with high numbers of suspected specimens. We recently reported differences in the seasonality of influenza and other respiratory viruses in Cameroon^10^ with higher circulation of rhinoviruses from January to June.

The aim of this study was to evaluate the performance of the SD Bioline RAT using the rRT-PCR as the standard reference. This was carried out with the intention of selecting a suitable tool for investigating potential outbreaks of ILI in a resource-limited country. The results obtained from our study showed excellent specificity and PPV, (100%) and no false-positive results were identified. Very low sensitivities (29·4%) and NPV (89·2%) indicating that there were false-negative results by RAT obtained. Previous studies testing RATs reported a higher sensitivity in the range of 44–77% and specificities of 96·8–100%.^2^,^11^ Thus, the specificity described in our study was comparable to the specificities previously reported. Unlike previous reports, our data show very low sensitivity. As previously reported, high viral titres increased the chance of detecting viruses using RAT.^12^,^13^ Therefore, low viral titres most likely explain this decline in sensitivity observed, considering that the proportion of patients with low viral titres was high among rRT-PCR positive patients (Figure 1). The cause of this low viral titres is currently not known, but this may have been due to prolonged time from illness onset to specimen collection. Results from other studies^11^ suggest that the delay as the onset of symptoms to the specimen collection is an important factor driving test sensitivity. Potentially, this delay might have decreased viral antigen levels in respiratory tracts secretions, decreasing the sensitivity of the RAT. Another reason for the low sensitivity could be due to the variation in test-collection method by multiple practitioners. It has been previously reported that test-collection method may be an important factor for the accuracy of RAT.^14^ Clearly, all conditions above may be exacerbated in resource-limited countries. In this study, we have also demonstrated a statistically significant effect of age on RAT sensitivity (*P* = 0·05), with increasing sensitivity in the younger age groups. The sensitivity of RAT has been reported to be higher in children compared with adults due to higher quantities of viral shedding.^15^ However, these are simply preliminary results considering that data presented were collected from only one site.

Our results indicate that SD Bioline could be used to screen influenza infections in a resource-limited setting lacking laboratory capabilities to identify influenza by culture or molecular techniques during an outbreak (PPV = 100%). These data also suggest that negative RAT should be confirmed using other laboratory tests such as RT-PCR to conclude. RATs with improved sensitivities are thus still needed for the efficient diagnosis of ILI in resource-limited settings.
